# Managing Addiction and Overdose Deaths: The Debate Over America’s Safe Injection Spaces

**DOI:** 10.7759/cureus.50406

**Published:** 2023-12-12

**Authors:** Farhaad Rasool, Arthur A Klein, Joerg R Leheste

**Affiliations:** 1 Biomedical Sciences, New York Institute of Technology (NYIT) College of Osteopathic Medicine, Old Westbury, USA; 2 Osteopathic Medicine, New York Institute of Technology (NYIT) College of Osteopathic Medicine, Old Westbury, USA

**Keywords:** hepatitis and hiv, blood-borne diseases, united states congress, covid-19 pandemic, opioid crisis, saving lives, overdose risk reduction, opioid overdoses, supervised injection sites, drug addiction

## Abstract

In the decade between 2010 and 2020, the number of people killed by opioid overdoses more than tripled, reaching 68,630. Among drug users in their second and third decades of life, hepatitis B and C are also on the rise. New York City's two new supervised drug injection sites (SDISs) reversed 114 overdoses within two months, and 585 people injecting drugs visited the facility 4,974 times. By providing medical professionals and a sterile and safe environment, supervised injection sites reduce overdose risk and save lives. This suggests that SDISs could be an important adjunct to currently failing strategies to combat the nation's raging opioid crisis, particularly in the aftermath of the COVID-19 pandemic. Here, we examine the perception and impact of SDISs as well as efforts by the United States Congress (H.R. 7029 and H.R. 6159) toward their prevention. We look into the perspectives of major stakeholders, such as residents, business owners, drug users, legislators, taxpayers, and the general public, and investigate the short- and long-term consequences of SDISs based on crime statistics and published data on opioid use, overdose deaths, and blood-borne disease transmission rates.

## Introduction and background

Unsupervised drug injections present a substantial public health hazard within the United States, thereby complicating efforts to stem the escalating tide of illicit drug consumption and overdose fatalities. The alarming statistics surrounding drug overdose fatalities indicate the loss of more than 932,000 people since 1999 [1-2], highlighting the seriousness of this issue as well as the need for action. The opening of the first supervised drug injection sites (SDISs) in New York City (NYC) in November 2021 has sparked heated debate about whether such facilities can provide a practical solution to the country's overdose crisis.

On March 3, 2023, the Defund Heroin Injection Centers Act of 2023 (H.R. 1355) was introduced to the U.S. House of Representatives, initiating a contentious and prolonged deliberation that delves into ethical and legal considerations. At the core of this discussion lies the inquiry into whether SDISs could unintentionally promote or exacerbate substance abuse, thereby adding to the complexity of the issue and undermining current efforts to develop efficacious interventions.

The opioid epidemic, which has been identified as the primary catalyst for the concerning increase in drug-related fatalities, has caused a substantial surge in overdose deaths ever since the COVID-19 pandemic broke out in 2019. During the year 2020, there was a notable surge in opioid-related fatalities across the nation, with 68,630 occurring as opposed to 49,860 the previous year. This represents an alarming increase of 38% in the total number of fatalities [1-2]. The most recent data for 2021 indicates another increase to 80,411 lives lost or 16.8%. The compounding effects of the pandemic have been linked to a general rise in drug usage rates, according to research on this phenomenon [1-2].

Although there was a general rise in opioid-related fatalities among all racial groups, with the largest increase occurring among the white American population, the COVID-19 pandemic disproportionately affected minorities and indigenous communities (Black, Hispanic, Asian, Native Hawaiian/Pacific Islander, and American Indian). This is especially concerning for those communities coping with the consequences of a catastrophic crisis in which opioid-related fatalities have impacted their members disproportionately [3]. SDISs are designed to combat the problem of opioid-related overdose deaths, which have reached an all-time high and require a reassessment of current preventive measures by lawmakers [3].

Despite an extensive array of preventive measures already in place, opioid overdoses accounted for about three-quarters of all 106,699 American drug-related deaths in 2021 [1]. This grim reality underscores the urgent need for innovative, creative, and cost-effective solutions to tackle the problem. The proposition of SDISs as a potential strategy is met with intense scrutiny, emphasizing the necessity for a comprehensive evaluation involving all major stakeholders. As the debate surrounding the Defund Heroin Injection Centers Act unfolds, the nation grapples with the imperative to chart a course that effectively addresses the multifaceted challenges posed by unsupervised drug injections.

## Review

Review strategy

This scoping health policy review is based on the systematic analysis and interpretation of articles associated with the National Library of Medicine (PubMed), Google Scholar, Scopus (Elsevier), and other resources vetted by the principal investigator (PI), such as select foundations (e.g., Kaiser Family Foundation, Pew Research Center), government agencies (e.g., Library of Congress, U.S. Department of Justice (DOJ), U.S. Food and Drug Administration, Centers for Disease Control and Prevention), municipalities (e.g., New York City (NYC), Vancouver), and news agencies (e.g., CNN, The New York Times, The Wall Street Journal, Global Newswire). Articles and reports were chosen and assessed based on their overall relevance to the research topic, credibility, and significance. The following search terms and expressions were used but were not limited to "opioid crisis, supervised drug injection sites, people who inject drugs, PWID, drug overdose deaths, and "transmissible blood-borne infections." The research was conducted over a 12-month period.

Major stakeholders

A variety of parties are involved in the establishment and operation of an SDIS through collaborative efforts. In regard to licensing and funding, governmental entities, including health departments and municipal governments, assume a critical function. Healthcare practitioners, such as physicians and nurses, deliver vital medical services and oversight. Community organizations and non-profits not only operate these facilities and advocate for them but also deal with local issues. Gathering information from drug users can be essential in determining the adequacy of services in terms of effectiveness, safety, and accessibility. Collaboration between law enforcement, local businesses, their staff, and community members is essential to guaranteeing public safety as well as the appropriate implementation of harm reduction strategies. In order for the general public and their legislative representatives to be informed and to allocate resources as efficiently as possible, researchers in the fields of public health and health policy will gather, evaluate, and distribute data. The effectiveness of an SDIS depends on these stakeholders working together in a productive way, which can vary depending on the situation, the site, and the political climate.

It is plausible that residents, their families, local businesses, and their customers near an SDIS may object to the illegal drug use and other substance activities that are taking place at or near their homes and properties. This has been evidenced by community activism against SDISs in NYC and so-called landscapes of antagonism against local syringe service programs (SSPs) in California's Central Valley [4,5]. Other members of the community may have different opinions given that unregulated and medically supervised injection sites are replacing random public spaces like sidewalks, parks, and transportation hubs as the locations for illicit drug use and unsanitary injection waste [6]. According to research conducted in Australia and Canada, the availability of injection sites can significantly lower the rate of random public injection in impacted areas [6,7]. One of the primary issues that these facilities are designed to address is the prevalence of overdose deaths. Two months after the opening of both New York locations in 2021, 585 registered users of illegal drugs had frequented the facilities 4,974 times, preventing an estimated 114 overdoses [8].

This strategy may also be of significant value to local business owners and hospitality professionals. According to the accounts of illicit drug users in NYC, public restrooms are one of the most frequently used locations for drug injection [9]. Therefore, employees in the service industry are often the first to encounter and deal with drug overdoses in these bathrooms [9]. By opening additional injection sites, more drug users could be redirected from using public restrooms to supervised locations with trained staff, taking the burden off the shoulders of hospitality workers untrained for such incidents.

People who inject drugs (PWID) illegally and now have access to safe injection sites benefit greatly from the opening of drug injection facilities as the sharing of contaminated injection equipment is directly linked to HIV/AIDS, hepatitis B, and hepatitis C [10]. This is because users frequently share "dirty" needles in uncontrolled settings where diseases can spread quickly. Contrarily, controlled injection sites offer simple access to sterile syringes and medical professionals, lowering the risk of needle sharing and disease transmission. One of the most important effects of drug injection sites is the ability of trained healthcare professionals to stop fatal overdoses before they happen by having oxygen and naloxone on hand.

Policymakers are another important stakeholder group that may benefit or suffer from the presence of supervised injection sites, depending on their position and that of their constituents. Legislators are likely to support these sites if research can conclusively demonstrate that they deliver on their promises and are valuable resources in the fight against the opioid epidemic. Without concrete evidence, taking a supportive stance may be interpreted as a call to drug abuse and a waste of tax dollars. These dynamics are evident at the aforementioned foreign locations. Successful drug injection site pilot programs have been implemented in Sydney and Vancouver to reduce public injections, encourage drug detoxification, and provide sterile needles to stop the spread of disease [6]. However, changes in government leadership posed a threat to both locations [6]. Policymakers are frequently conflicted about legalizing drug use as a means of advancing harm reduction and controlling the opioid epidemic from an ethical standpoint. However, the issue at hand is complex, and new alliances and innovative approaches may provide viable solutions.

Current policy and challenges

In order to combat the crack epidemic, Congress passed the Anti-Drug Abuse Act of 1986, which included the "Crack House Statute" (21 U.S. Code Section 856). This law, which has since been updated, was intended to place restrictions on any facilities that supported the use or production of illegal drugs [11]. The existence of SDISs violates this statute. As a matter of fact, in 2018, Deputy Attorney General Rod Rosenstein made it clear that "cities and states should expect the DOJ to meet the opening of any injection site with swift and aggressive action [12]." Despite this warning, two locations in NYC's East Harlem and Washington Heights have since opened. Funding comes from NYC and private sources for two nonprofit organizations, the New York Harm Reduction Educators and the Washington Heights Corner Project. Both organizations merged in 2022 to form OnPoint NYC [13]. The fact that any drug, whether legal or illegal, is allowed on the premises of injection facilities is one of the most contentious aspects of the current policy in this area. This allows extremely potent drugs like heroin and fentanyl to be purchased and used without fear of reprisal. These substances are also highly addictive, and PWID may develop lifelong addiction illnesses while under the protective status of the site. This, along with other unintended consequences, is what fuels the debate.

Regulation of the drugs that are allowed in those facilities would be one particular policy mechanism to address this issue. The U.S. Drug Enforcement Administration (DEA) currently classifies drugs into schedules. Schedule I drugs are defined as “substances or chemicals with no currently accepted medical use and a high potential for abuse [14]." Examples of Schedule 1 drugs include heroin, lysergic acid diethylamide (LSD), marijuana (cannabis), and 3,4-methylenedioxymethamphetamine (ecstasy). Schedule 2 drugs are those that have a high potential for abuse and can lead to severe levels of psychological and physical dependence. These include drugs like fentanyl, cocaine, and methamphetamine. The imposition of constraints may reduce unintended consequences, but at the same time, it will also affect the impact of those sites that require careful consideration and calibration.

SDISs enabling drug use

As previously stated, the possibility that these sites promote drug use and serve as incubators for drug addiction is one of the most contentious and alarming concerns [15,16]. In spite of enforcing DEA drug schedule restrictions that defy the existence of supervised injection sites, regulations that promote and enforce safer injection practices, such as using clean needles and avoiding sharing injection equipment, can greatly enhance the sites' mission by reducing the spread of HIV and other blood-borne infections. For instance, a study found that over a 10-year period, a supervised injection site in Vancouver, Canada, saw a significant decline in the incidence of HIV among clients when restrictions on sharing injection equipment were implemented [17]. Reducing drug users' access to services has been associated with negative effects, such as a higher risk of overdosing. Clients who claimed they were turned away from the facility due to restrictions were found to be significantly more likely to report risky injecting behaviors and overdoses in the subsequent six months, according to a study conducted on a supervised drug injection site in Sydney, Australia [18]. Overall, the evidence on the effects of restrictive rules at SDISs is limited and mixed, and more research is needed to better understand the potential benefits and harms of these policies.

Requiring users to be actively enrolled in an addiction program in order to end their dependency could be one policy move that would help to lessen the negative perception of drug injection sites. This would enhance the goal of injection facilities, which is to offer drug users a secure and medically supervised environment in which to administer drugs as well as to find and assist those who are prepared to take action to overcome their addiction. Ultimately, this strategy may also increase acceptance by the public as well as by many lawmakers currently in opposition. According to reports from Europe, where supervised injection sites have been operational since the 1980s, the presence of such facilities has increased patient enrollment in drug treatment programs, lending credence to the preceding idea [7]. While the presence of injection sites may initially encourage drug use by providing a comfortable setting for injections, many users may also be prepared to seek out treatment and take action to overcome their addiction. Facilities in Sydney and Vancouver have demonstrated that they are efficient entry points for addiction treatment, counseling, and related services, supporting this idea [7]. In accordance with this concept, 38% of drug users at the location in Sydney were significantly more likely to begin treatment for their addiction compared to 21% of drug users not attending the site. At the Vancouver location, speaking to an addiction counselor was significantly associated with timely enrollment in detoxication services [5]. Results so far suggest that by extending the scope of injection sites to include counseling and addiction treatment, overall drug use may be reduced, validating the initial concept of SDISs. For SDISs to be validated or refuted, more extensive research will be needed to overcome the small sample size limitations.

Methadone programs versus SDISs

While SDISs and methadone programs are interrelated and both geared toward harm reduction for those struggling with opioid addiction, they are also distinct and not interchangeable. The most important differences lie in location and scope. Methadone programs function as maintenance treatments conducted in specialized clinics focused on withdrawal symptoms and cravings to be alleviated using the synthetic opioid methadone which is predominantly consumed orally. The main objective of this approach is to enhance the ability of individuals to function with the help of strictly supervised medication administration. This approach has been used in the treatment of addiction since the 1960s [19]. On the other hand, SDISs function as harm reduction facilities in which PWID self-administer mostly injected, pre-obtained substances while being monitored by medically trained staff. These facilities are located within community environments, in which these substances would otherwise be consumed in an uncontrolled fashion. Therefore, the focus of SDISs lies in the mitigation of imminent hazards linked to substance use, including disease transmission, and overdose. Metabolic programs prioritize structured treatment. Although both approaches adhere to unique methodologies, they share a common objective of reducing harm. The current situation emphasizes the necessity of employing a combination of sequential and parallel strategies that mutually inform one another in order to effectively address an expanding issue.

Additional medical screening opportunities at SDISs

Offering medical entry screening for each person who wishes to use the site is another intriguing possibility for injection sites beyond curing addiction. Any blood-borne illnesses, such as HIV or hepatitis B and C, would be screened for. Patients could be referred right away for disease management in the event of an unfavorable result. The hepatitis C epidemic affects PWID the most severely, but research shows that these individuals are also the least likely to receive treatment [20]. In fact, it is estimated that millions of American intravenous drug users are infected with hepatitis C and require access to care [20]. An initial screening may include an assessment of the patient's medications for potential drug-drug interactions that could occur with illegal drug use. By diagnosing, treating, and preventing disease at supervised injection sites, an improved standard of care for people affected by drug addiction could be offered.

The Defund Heroin Injection Centers Act of 2023

The Defund Heroin Injection Centers Act of 2023 (H.R. 1355), submitted to the 118th Congress, prohibits federal funding of any state, local, tribal, or private entity operating supervised injection centers [21]. Although the sites in New York are operated without federal assistance, the bill challenges the potential expansion of the sites into other U.S. metropolises such as San Francisco, Philadelphia, and Denver [7,22]. Adoption into law would serve as an additional barrier to planned and existing sites without consideration of workable alternatives. The bill is consistent with the "war on drugs" approach intended to reduce drug use, trafficking, and distribution by toughening the prison terms for offenders. President Nixon issued the first proclamation of the ongoing drug war in 1971 [23]. Unfortunately, the “war on drugs” continues to have a disproportionately negative impact on minorities. According to 2016 data, nearly 60% of inmates serving time in state prisons for drug offenses and 80% of inmates serving time in federal prisons are Black or Latino [24]. Despite its disproportionately negative impact on minorities, the "war on drugs" slogan has been used politically to win over the public. After the DOJ hinted that it might be willing to consider supervised injection sites, the Stop Injection Sites for Illegal Drugs Act of 2022 (H.R. 7029; 117th Congress) was introduced [25]. A spokesperson stated that they were evaluating facilities and discussing appropriate guardrails with regulators [21]. When the Biden administration first took office, the DOJ did not take a position on injection sites but now declares that they are considering their use "as part of an overall approach to harm reduction and public safety [26]."

Unsafe drug injection practices and blood-borne diseases

Without access to SDISs, PWID would no longer be in a sterile environment and would be at a higher risk of contracting serious diseases. Due to drug injection with contaminated needles, one of the most lethal consequences of the opioid crisis is an increase in blood-borne infections, including hepatitis B and C, HIV, and bacterial endocarditis [27]. HIV and hepatitis C are frequently transmitted this way, both of which cause serious health problems for both people and the environment. According to estimates, 1.2 million Americans are thought to be HIV positive, with about 13% of them being completely unaware of their condition [28]. PWID accounts for 7% of new HIV infections in the U.S. [28]. This percentage has decreased throughout the past decades due to the introduction of SSPs and prevention resources.

**Figure 1:**
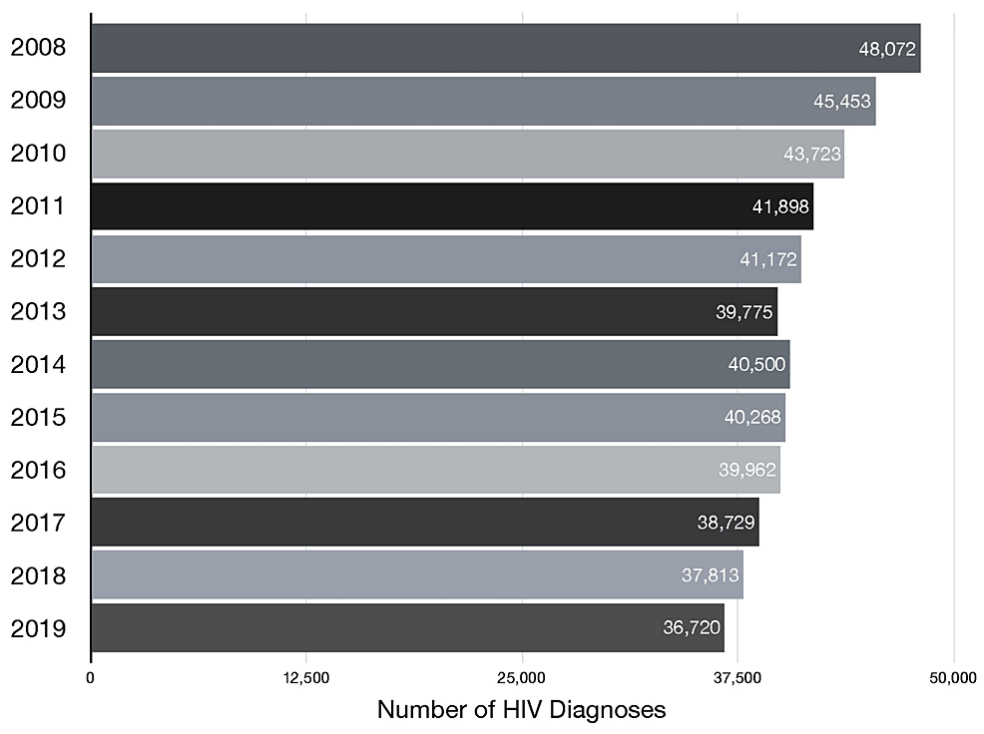
New U.S. HIV diagnoses (2008-2019) Between 2008 and 2019, the number of new HIV diagnoses dropped by more than 11,000 cases [29]. This decline can be attributed to needle exchange programs, improved HIV prevention education, and anti-retroviral medications. Despite a decrease in the number of new cases, many continue to occur as a result of drug injections using contaminated or shared needles. While data from the COVID-19 pandemic appears to support this trend, the sharp drop in testing due to the pandemic, which disrupted clinical care and community services, complicates a conclusive analysis.

While HIV infections have been declining in the United States, hepatitis B and C are on the rise. The decrease in new HIV cases is accompanied by needle exchange programs, improved education on HIV prevention, and anti-retroviral medication to manage the disease (Figure 1).

**Figure 2:**
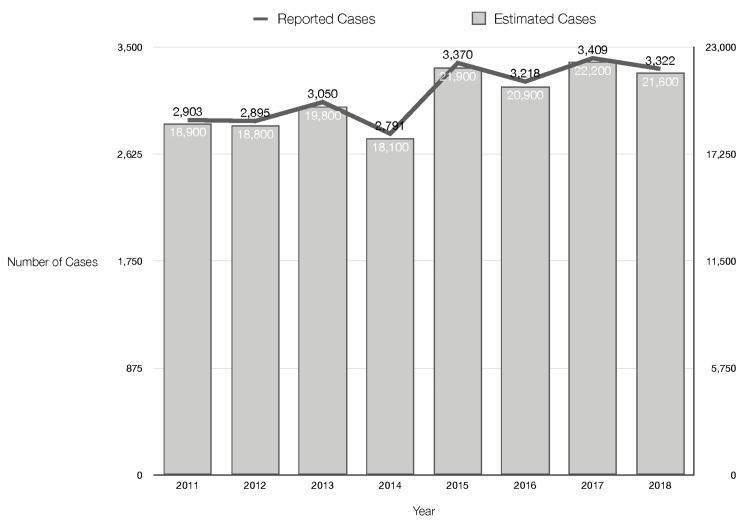
Reported vs. estimated U.S. hepatitis B cases While the number of reported hepatitis B cases has increased slightly in recent years, estimates are more than six times higher [31]. According to the CDC, many people who have hepatitis B are unaware of their infection, which promotes disease spread. Hepatitis B is a vaccine-preventable disease; individuals can also seek treatment, including various antiviral drug options.

Hepatitis C is estimated to affect 2.4 million people in the United States, but the true figure could be closer to 4.7 million [30]. Furthermore, an estimated 850,000 people have hepatitis B, with the actual number possibly approaching 2.2 million (Figure 2) [31].

**Figure 3:**
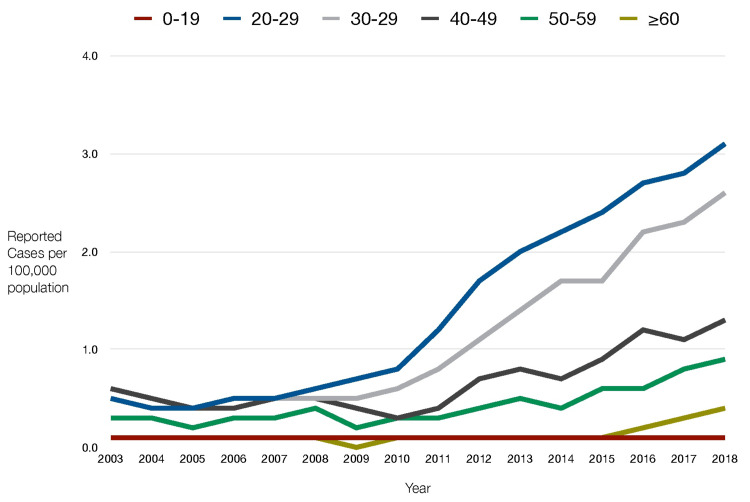
Reported acute U.S. hepatitis C cases by age From 2008 to 2018, the age groups 20-29 and 30-39 had the highest rates of hepatitis C [32]. Research shows that the most common drug users are also between the ages of 20 and 40. Reports confirm that the most common cause of hepatitis C infection is intravenous drug use. The numbers document a rise in hepatitis C in all age groups over the last decade, except for those aged 0 to 19.

**Figure 4:**
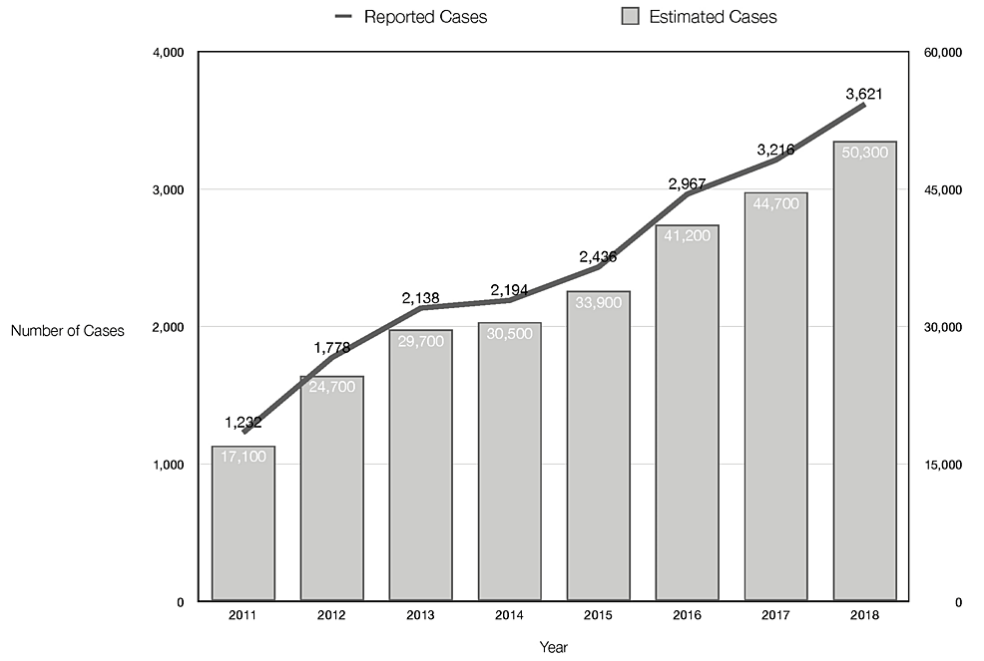
Reported vs. estimated U.S. hepatitis C cases Hepatitis C cases have increased exponentially in recent years, owing primarily to increased injection drug use. The estimated number of cases in 2018 was more than 14 times higher than the reported number [33]. While there is no vaccine to prevent hepatitis C, there are medications available for those who are infected. Identifying individuals with the disease earlier may allow for better disease prevention.

This is because 51% of hepatitis C patients and nearly 67% of hepatitis B patients are unaware of their infection, increasing the risk of transmission (Figures 3-4) [30,32,33].

According to research conducted with a PWID cohort in Massachusetts, more than half of PWIDs under the age of 25 report sharing needles [34]. This finding is consistent with the fact that in 2018, millennials (people in their 20s and 30s) accounted for 36.5% of newly reported chronic hepatitis C infections (Figure 4) [30,31]. In fact, the rate of hepatitis C infections reported to the CDC in 2018 was four times higher than in 2010. According to the Office of Infectious Disease and HIV/AIDS Policy, "2018 marked a decade of increases in new hepatitis C infections among people in their twenties and thirties, with injection drug use as the primary mode of transmission [27]." The analyses in the report support the link between age, drug use, and blood-borne infections. The reason why new HIV diagnoses are decreasing but new hepatitis B and C diagnoses are trending upward may have to do with the fact that there are more people unaware of their hepatitis B/C diagnosis (51% and 67%, respectively) versus their HIV diagnosis (13%); because of this, less treatment is sought for hepatitis B and C, and thus these diseases continue to spread.

Analysts have reported an increase in substance use and drug overdoses in the United States as a result of the recent COVID-19 pandemic [35]. Analysis indicates that this encompasses a 30% increase in drug overdoses between 2019 and 2020, coinciding with the start of the pandemic [1]. This number encompasses all drugs, with the majority being opioid-related (75.4%) and a rising number of psychostimulants, including methamphetamine. According to addiction researchers, stress, trauma, and mental distress have all been linked to an increased risk of developing a substance use disorder. Experts also report that pandemic-related factors such as social isolation and stress, individuals using drugs alone, and limited access to substance abuse treatment have increased the frequency of drug overdoses and, inevitably, blood-borne diseases [35]. Data from the Transformed Medicaid Statistical Information System from 2019 to 2020 show that states that permitted community-based operation of SSPs had lower HIV transmission in PWID during the COVID-19 pandemic [36,37]. These findings provide a clear mandate for state and federal legislation in support of SSPs and SDISs.

Even the transmission of viral diseases such as Ebola, Lassa fever, and malaria has been linked to unsafe needle injections. The vast majority of those cases, however, have been the result of inadequate hygiene and medical training during therapeutic injections in developing-world hospitals outside of the United States [38].

Stakeholders opposing the Defund Heroin Injection Centers Act of 2023

The primary coalition opposing congressional actions against SDISs is made up of PWID, their families, and advocacy groups. A user at SDISs is not subject to law enforcement arrest, is not exposed to potentially contaminated needles, and is not at risk of overdosing on opioids. In an interview with the New York Times, crack user Chynna Rodriguez expressed gratitude for the site, explaining, "We're here, the cops aren't bothering us, we're not dying in the park, and we're not out in front of kids." When it comes to PWID, the perceived benefits appear to revolve around safety and privacy.

The majority of hospitality and service industry workers are likely to oppose H.R. 1355 because they will be directly impacted if sites do not receive funding and cannot open. According to drug users, public restrooms are one of the most common places where drugs are injected [9,10]. In the absence of a private space to inject drugs, users will most likely resort to using public spaces, such as public restrooms. Service workers are impacted because they are frequently the first to respond to such activity, which can be upsetting and dangerous. A study based on semi-structured qualitative interviews with 15 service industry workers conducted via convenience sampling throughout NYC found that 93% had encountered drug use in their business bathroom, with three encountering unresponsive drug users [9,10]. Furthermore, 73% reported physical syringes left behind, posing health risks. Ninety-three percent of the same cohort agreed that supervised injection sites would reduce the number of people using business restrooms to inject drugs. They also believed that "not in my backyard" arguments from community boards could serve as a roadblock to the operation of SDISs.

Finally, healthcare workers who see SDISs as a way to combat the opioid epidemic in the United States are likely to oppose congressional attempts to interfere with SDISs. Many people believe that the primary goal of these sites is to prevent overdose deaths and that by providing healthcare workers with naloxone and other reversal drugs, they are accomplishing this. Former NYC health commissioner Dr. Dave A. Chokshi, MD, stated: "We feel a deep conviction and also a sense of urgency in opening overdose prevention centers [39]." According to a landmark study on the efficacy of unlicensed safe consumption sites in America, there were no overdose deaths at one specific site from 2014 to 2019 [40]. Over 10,000 injections had been completed during this time period, with 33 resulting in an opioid overdose. All 33 overdoses were successfully treated with naloxone, demonstrating that these sites aid in the prevention of drug overdose deaths, which are a serious issue in the United States today.

Stakeholders supporting the Defund Heroin Injection Centers Act of 2023

Communities are divided on this issue because, while SDISs may be beneficial, many people do not want them near their homes or public spaces. The first point of contention among members is why their community was chosen to host a supervised injection site. The community board members are concerned that opening a supervised drug injection site will further stigmatize the community as a place where drug sales and use are tolerated. In New York, for example, one location opened in East Harlem, which already has several drug treatment centers. Jeffrey Mays and Andy Newman of the New York Times reported that various community boards have protested against these substance abuse facilities. For example, Syderia Asberry-Chreshfield, co-founder of the Greater Harlem Coalition, stated: “Not only can I buy my drugs here, but I can safely shoot them up in a comfortable atmosphere where people are watching over me? And then they go outside and they wreak havoc in the neighborhood. We can’t live like this.” [41]. However, research at new sites in Vancouver and Sydney has found little negative impact on the surrounding communities [6,7]. While evidence points in both directions, it is clear that community members do not share the same perspectives as service industry workers working in the same community. The question then becomes: what are the priorities, and how can a workable compromise be reached that takes into account the interests of all stakeholders? At the moment, it appears that no middle ground has been established, which is why the subject is so divisive.

## Conclusions

SDISs provide a scientifically supported solution to the opioid epidemic by creating a safe environment for users, reducing blood-borne diseases, and preventing overdose deaths. However, the underlying issue is much broader and more complex, and while SDISs can be an important component in saving lives, they will not be sufficient to end the opioid epidemic and its devastating consequences for individuals, their families, communities, and American society as a whole. Former US President Richard Nixon launched the War on Drugs in the early 1970s, but it failed. Instead of eradicating the drug trade in America, it has unfairly vilified and marginalized African-American and Hispanic communities, exacerbating intergroup conflict and suffering. While a comprehensive strategy is currently lacking, the U.S. Congress and state governments should instead foster consensus and unity in the fight against American drug deaths. This will necessitate complete transparency among all stakeholders, as well as the prudent testing and implementation of novel ideas and strategies. Congressional bills, such as the Defund Heroin Injection Centers Act of 2023, highlight the controversies surrounding this topic and the need for more research to better understand medical and community outcomes to make better and more informed decisions in the future.
